# A comparative investigation of normal and inverted exchange bias effect for magnetic fluid hyperthermia applications

**DOI:** 10.1038/s41598-020-75669-3

**Published:** 2020-10-29

**Authors:** S. P. Tsopoe, C. Borgohain, Rushikesh Fopase, Lalit M. Pandey, J. P. Borah

**Affiliations:** 1 Department of Physics, National Institute of Technology Nagaland, Dimapur, Nagaland 797103 India; 2 Central Instrumentation Facility (CIF), Indian Institute of Technology Guwahati, Guwahati, 781039 India; 3 Bio-Interface & Environmental Engineering Lab, Department of Biosciences and Bioengineering, Indian Institute of Technology Guwahati, Guwahati, Assam 781039 India

**Keywords:** Cancer, Materials science, Nanoscience and technology, Physics

## Abstract

Exchange bias (EB) of magnetic nanoparticles (MNPs) in the nanoscale regime has been extensively studied by researchers, which have opened up a novel approach in tuning the magnetic anisotropy properties of magnetic nanoparticles (MNPs) in prospective application of biomedical research such as magnetic hyperthermia. In this work, we report a comparative study on the effect of magnetic EB of normal and inverted core@shell (CS) nanostructures and its influence on the heating efficiency by synthesizing Antiferromagnetic (AFM) NiO (N) and Ferrimagnetic (FiM) Fe_3_O_4_ (F). The formation of CS structures for both systems is clearly authenticated by XRD and HRTEM analyses. The magnetic properties were extensively studied by Vibrating Sample Magnetometer (VSM). We reported that the inverted CS NiO@Fe_3_O_4_ (NF) MNPs have shown a greater EB owing to higher uncompensated spins at the interface of the AFM, in comparison to the normal CS Fe_3_O_4_@NiO (FN) MNPs. Both the CS systems have shown higher SAR values in comparison to the single-phased F owing to the EB coupling at the interface. However, the higher surface anisotropy of F shell with more EB field for NF enhanced the SAR value as compared to FN system. The EB coupling is hindered at higher concentrations of NF MNPs because of the enhanced dipolar interactions (agglomeration of nanoparticles). Both the CS systems reach to the hyperthermia temperature within 10 min. The cyto-compatibility analysis resulted in the excellent cell viability (> 75%) for 3 days in the presence of the synthesized NPs upto 1 mg/ml. These observations endorsed the suitability of CS nanoassemblies for magnetic fluid hyperthermia applications.

## Introduction

Over the past few decades, the tremendous focus has been placed on biomedical research. Among all the health diseases, Cancer has become a major public health problem in our present world. Even after inventing multiple techniques for cancer treatment, the rapid growth of cancer is effectively underway worldwide. According to the International Agency for Research on Cancer (IARC), the global cancer chaos was estimated as 18.1 million new cases and 9.6 million deaths in the year 2018^1^. One of the emerging techniques to treat the rapid growth of cancer is magnetic fluid hyperthermia (MFH). It is a process design in treating cancer by means of generating heat by the MNPs during the magnetization reversal process under the environment of an alternating magnetic field^2–5^. However, this application has certain restrictions which impose upper limits of process variables. The heating process is valid for suitable ranges of frequency and applied field for hyperthermia treatment criterion given by Hergt^5^. The power loss generated within the MNPs on exposing to alternating magnetic field comprises of hysteresis, eddy current and residual losses (Néel’s relaxation and Brownian relaxation), which truly influence and determine the Specific absorption rate (SAR) of the MNPs^6^. In order to advance hyperthermia treatment, the SAR value should be greatly enhanced within the field and frequency range of clinical limit so that several potential side effects on the healthy body tissues could be avoided. The SAR of MNPs depends on several factors such as size distribution of the MNPs, frequency and amplitude of the applied field, magnetization, magnetic anisotropy and particle–particle interaction^7–10^. Presently, researchers are putting more emphasis on enhancing the SAR value through the bi-magnetic CS NPs with two different magnetic phases. This displays an exchange bias (EB) coupling phenomenon between the core and shell materials which results in tuning several magnetic properties of the NPs^11–14^. EB effect for a particular system consisting of AFM and FiM/FM materials arises when uncompensated AFM spins are usually pinned up with the spins of FiM phase at the magnetically interface surface,which in turn leads to hysteresis loop shift and amplification of coercivity^15^. EB coupling of its first kind was experimentally found in 1956 on a CS Co(FM)@CoO(AFM) by Meiklejohn et al. with Curie temperature (T_C_) higher than the AFM Néel temperature (T_N_)^16,17^. Recently, immense attention has been put forth on researchers with unusual configurations of “single inverted” and “double-inverted” CS system. A single inverted is an unusual structure or a conflicting version of the normal CS system, where the core material is composed of AFM phase and shell material of FM/FiM phases such as NiO@Ni_x_Co_1−x_O^18^, FeO@Fe_3_O_4_^19^. However, in a single inverted system, the Néel temperature is smaller than the Curie temperature (T_N_ < T_C_) similar to the condition of the usual CS system. On the other hand, a doubly inverted system like MnO@Mn_3_O_4_^20,21^ comprises of an AFM Néel temperature higher than the Curie temperature (T_N_ ˃ T_C_) in contradiction to the conventional CS system. Recently, immense attention has been driven by researchers approaching a theoretical perspective for better understanding on the mechanism of EB phenomenon and its properties through a powerful Metropolis algorithm of Monte Carlo simulation for both single inverted^22^ and doubly inverted CS^23,24^ systems. Many researchers have also explored an exchange coupling based on soft and hard material CS system for magnetic hyperthermia^24–28^. Phadatare et al. reported that CS of CoFe_2_O_4_@Ni_0.5_Zn_0.5_Fe_2_O_4_ resulted in enhanced SAR due to exchange coupling at the interface which is attributed to tuning the optimal magneto crystalline anisotropy^29^. However, hard and soft ferrite based magnetic CS is not favourable for hyperthermia due to Co content in the hard ferrite based materials which is toxic and harmful for the human body. Recently, Vikas et al. confirmed that an exchange coupling between two soft magnetic ferrite leads to increase in the magnetic susceptibility and anisotropy of the CS MNPs compared to the single phase counterparts resulting to the enhancement of SAR value up to 827 W/g, which is about 9 times greater than the conventional ferrite based MNPs^30^. Also, for CS MNPs, manipulating the thickness of the core and shell displays a very important role in tuning the magnetic properties via the exchange coupling between the two MNPs. Robles et al. experimentally showed those magnetic properties (saturation magnetization, anisotropy,) and heating efficiency changes when the core and shell sizes are varied. Also, it was reported that the variation in the shell thickness has a greater impact on the magnetic response and heating efficiency compared to the variation in core size of the CS nanostructures^31^. Although researchers have extensively explored the exchange coupling phenomenon, there have not been many investigations on the influence of NPs heating ability due to EB effect. Further, hyperthermia studies based on normal CS system as well as inverted CS system of different combination of magnetic material have been explored, however, not much survey has been done so far on a comparative analysis of both normal CS and inverted CS nanostructures using the same materials.

In the present work, we have done a comparative study of magnetic properties and exchange coupling of a normal CS system with AFM N as a shell material and FiM F as the core over the properties of an inverted CS system with N as the core material and F as the shell to investigate as to which type of system will exhibit greater heating efficiency. We have also shown that higher MNPs heating ability or maximum SAR value is obtained when the EB coupling at their interface is higher. The synthesized MNPs are characterized physically and chemically. Magnetic and induction heating studies are performed to decipher the heating ability of the synthesized CS systems. Cyto-compatibility of the MNPs is also analysed to access the suitability for hyperthermia applications.

## Experimental

### Materials

The raw materials utilised in the synthesis of Fe_3_O_4_@NiO and NiO@Fe_3_O_4_ CS nanoparticle were purchased from merk and used without any further modification. The raw materials are Nickel chloride hexahydrate NiCl_2_·6H_2_O (99.9% purity), anhydrous iron chloride Fecl_3_ (97% purity) and sodium acetate CH_3_COONa (99% purity).

### Synthesis of inverted core/shell NiO@Fe_3_O_4_ and usual core/shell Fe_3_O_4_@NiO

The inverted CS NiO@Fe_3_O_4_ was prepared in a two-step reaction^32^. Firstly, NiO NPs was synthesized using the co-precipitation method which is also explained elsewhere^33^. 3.95 g of Nickel chloride hexahydrate (NiCl_2_·6H_2_O) was diluted in a solvent of 50 mL double-distilled water at room temperature to acquire a certain molar concentration. Then, the obtained solution was stirred magnetically at 50 °C for 40 min, followed by drop wise addition of 2.14 g NaOH dissolved in 10 mL of double-distilled water to the solution at a constant pH of 8. The product was green precipitate which was separated from the supernatant liquid and washed several times with the copious amount of ethanol and double distilled water to remove all the impurities. It was finally dried for 12 h at 80 °C. The dried sample was calcined for two hours at 500 °C to obtain the black coloured NiO NPs. The as-prepared NiO NPs was then used as the core in the synthesis of inverted CSNiO@Fe_3_O_4_ using a solvothermal method described elsewhere^34^. 1 g of the as-prepared NiO NPs was mixed in a solution of 5.44 g of sodium acetate (CH_3_COONa) and 4.20 g of Ferric chloride (FeCl_3_) in 50 mL of Ethylene glycol to obtain a homogeneous solution. The mixture was then continuously stirred for 30 min, after which the solution was inserted inside in a stainless steel autoclave and heat treated at 180 °C for 10 h. The resulting solution was allowed to cool properly at room temperature, after which a 15 min ultrasonication was carried out to ensure that a well disperse of NPs was formed. Finally, the product obtained was washed several times with double distilled water and ethanol, followed by centrifugation at 1500 rpm. A dark brownish precipitate of NiO@Fe_3_O_4_ was obtained after drying in the oven for 12 h at 80 °C. The final product was then grinded using an agate mortar and pestle to obtain a fine powder of NPs. The synthesis of the normal CS Fe_3_O_4_@NiO was done using a similar but reverse procedure explained above. Fe_3_O_4_ was synthesized first using the procedure described above and the product was used as the core in the synthesis of NiO shell as described above. The final product was dried at 80 °C and grinded to a fine powder of CS Fe_3_O_4_@NiO NPs.

### Characterization of the synthesized core@shell NPs

The crystallographic structure and phase identification are done using powder X-ray Diffractometer (XRD, Rigaku, ULTIMA IV) of wavelength (λ) = 1.5406 Å from 20° to 80° in 2θ range containing Cu-Kα radiation. Fourier Transform Infrared (FTIR, Agilent, Cary 630) spectrophotometer was utilized to characterize all different vibrational spectra of the as-prepared samples. The surface morphology and the particle size distribution were studied using high-resolution transmission electron microscopy (HRTEM, JEOL, JEM 2100). The magnetic field responses for all the samples are analyzed at three different temperatures (60 K, 200 K and 300 K) by using a Lakeshore 7410 vibrating sample magnetometer (VSM). The sample toxicity level and its biocompatibility study are done by MTT (3-(4,5-Dimethylthiazol-2-yl)-2,5-Diphenyltetrazolium Bromide) assay using human MG-63 cells^35,36^. Different sample concentrations were incubated with 10^4^ cells/ml in a CO_2_ incubator at 37 °C and 5% CO_2_ for 24 and 72 h before performing the MTT assay. The particle heating ability response for hyperthermia is examined at a fixed frequency and applied field (within clinical limit) using Easy Heat 8310 (Ambrell make UK) having 7 and 8 turns of diameter coils equipped with fiber optic sensor to measure the temperature. The induction heating study was performed at three different concentrations (0.5, 1 and 1.5 mg) suspended in 1 ml of double distilled water^37–39^. To avoid the agglomeration and settling, MNPs were sonicated right before performing induction heating experiment to ensure the proper dispersion in water^40^.All the experiments have been performed in triplicate and data are reported as average ± standard deviation.

## Results and discussion

### XRD analysis

**Figure 1:**
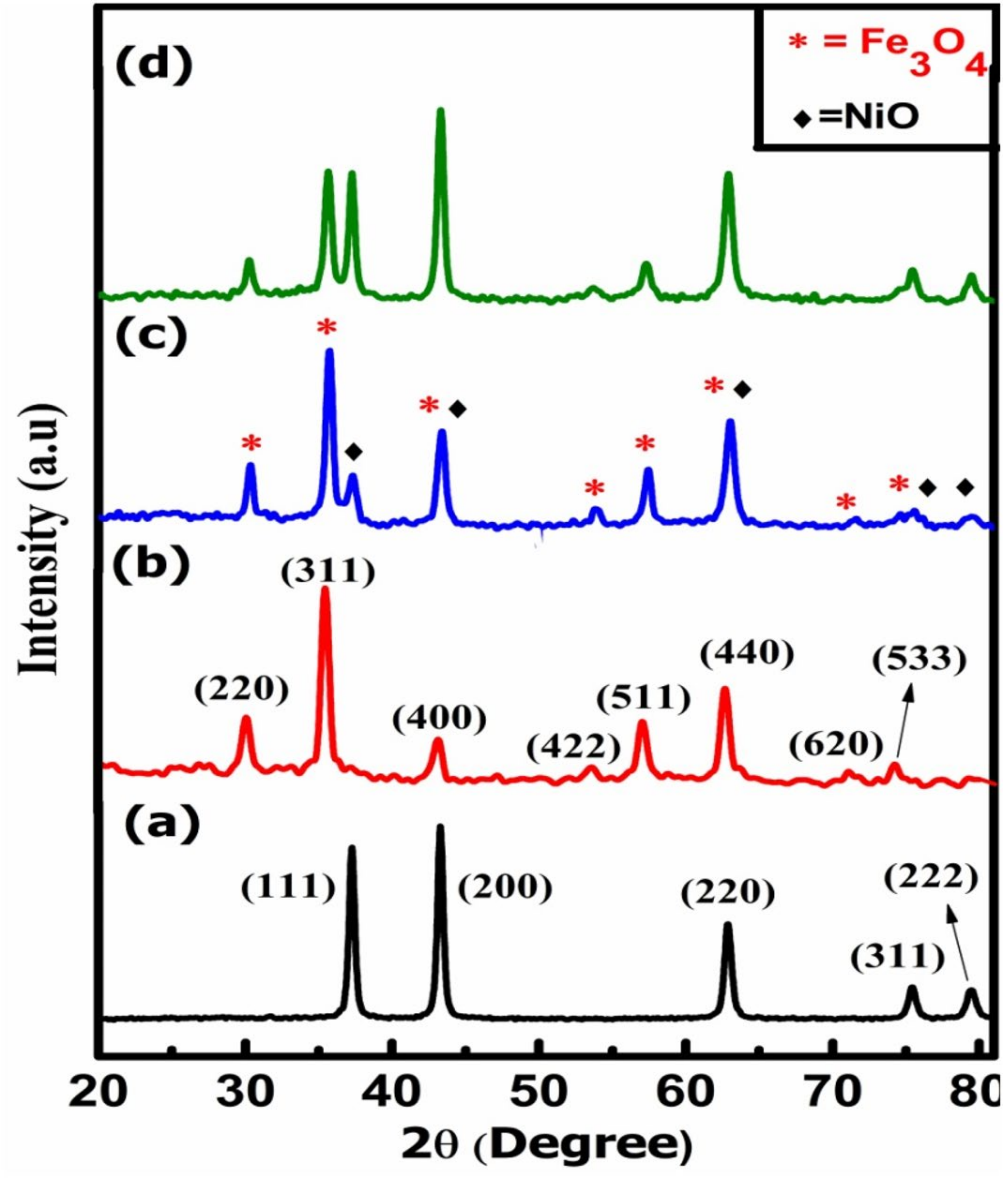
XRD patterns of (**a**) N, (**b**) F, (**c**) FN and (**d**) NF NPs.

Figure 1a–d shows the XRD spectrums of N, F and comparative spectrums of normal CS (NF) and inverted CS (FN). The co-existence of both N and F in the normal CS and inverted CS is clearly authenticated from the XRD pattern. The XRD results endorse a face-centred cubic structure with Fm $\stackrel{-}{3}$ m space group for N (JCPDS card no. 78-0643) and Fd $\stackrel{-}{3}$ m space group for F (JCPDS card no. 89-3854) respectively and no other impurity peaks were identified in the XRD crystallographic planes. The average crystallite size analysis is put through the full-width at half-maxima (FWHM) of the most intense peaks for N, F, NF and FN NPs by using the Scherer’s formula^41^. The crystallite size and lattice constant for all the samples are listed in Table 1. The crystallite sizes of shell MNPs are found to be comparable with bare NPs, while the crystallite sizes of core MNPs are reduced as compared to bare NPs. However, the formation of CS structures is not confirmed from the XRD and is further analysed using HRTEM. The lattice constant for bare N and F is in accordance with the reported values^42,43^. However, reduction in the lattice constant is encountered in the case of nanocomposite CS for FN and NF (Table 1). The decreasing notice in the lattice constant for the CS system may be attributed to the lattice dissimilarities between N and F respectively^44,45^.

**Table 1:** Crystallite size and lattice constant of NiO, Fe_3_O_4_, Fe_3_O_4_@NiO and NiO@Fe_3_O_4_.

Sample	Crystallite size (nm)		Lattice constant (Å)	
	D_N_	D_F_	a_N_	a_F_
N	17.60	–	4.177	–
F	–	18.58	–	8.393
FN	18.42	16.33	4.172	8.334
NF	16.76	18.21	4.175	8.345

### Fourier transform infrared analysis

**Figure 2:**
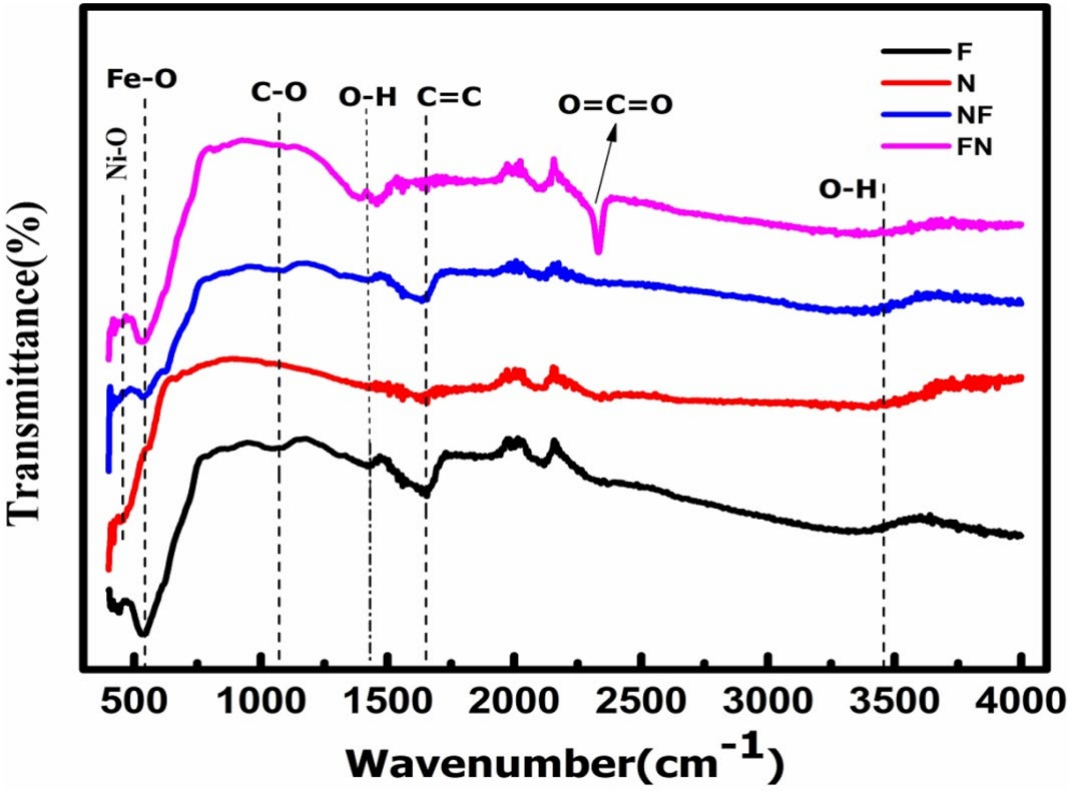
FTIR spectra of N, F, NF and FN NPs.

In order to understand the different vibrational characteristics of the absorption band of the crystal lattice, FTIR measurement is performed in the wave-number range of 450 cm^−1^ to 4000 cm^−1^ as depicted in Fig. 2. The frequency absorption band spotted at 2250 cm^−1^ signifies the presence of a strong double bond of O=C=O. The peaks observed at 1640 cm^−1^ and 1418 cm^−1^ correspond to C=O stretching and C–H bending vibration, respectively. These functional groups are originated from sodium acetate and ethylene glycol solvent used during the synthesis process. Finally, the IR bands at 541 cm^−1^ and 453 cm^−1^ confirmed the presence of Fe–O and Ni–O stretching vibration of both NiO and Fe_3_O_4_ respectively.

### SEM analysis

**Figure 3:**
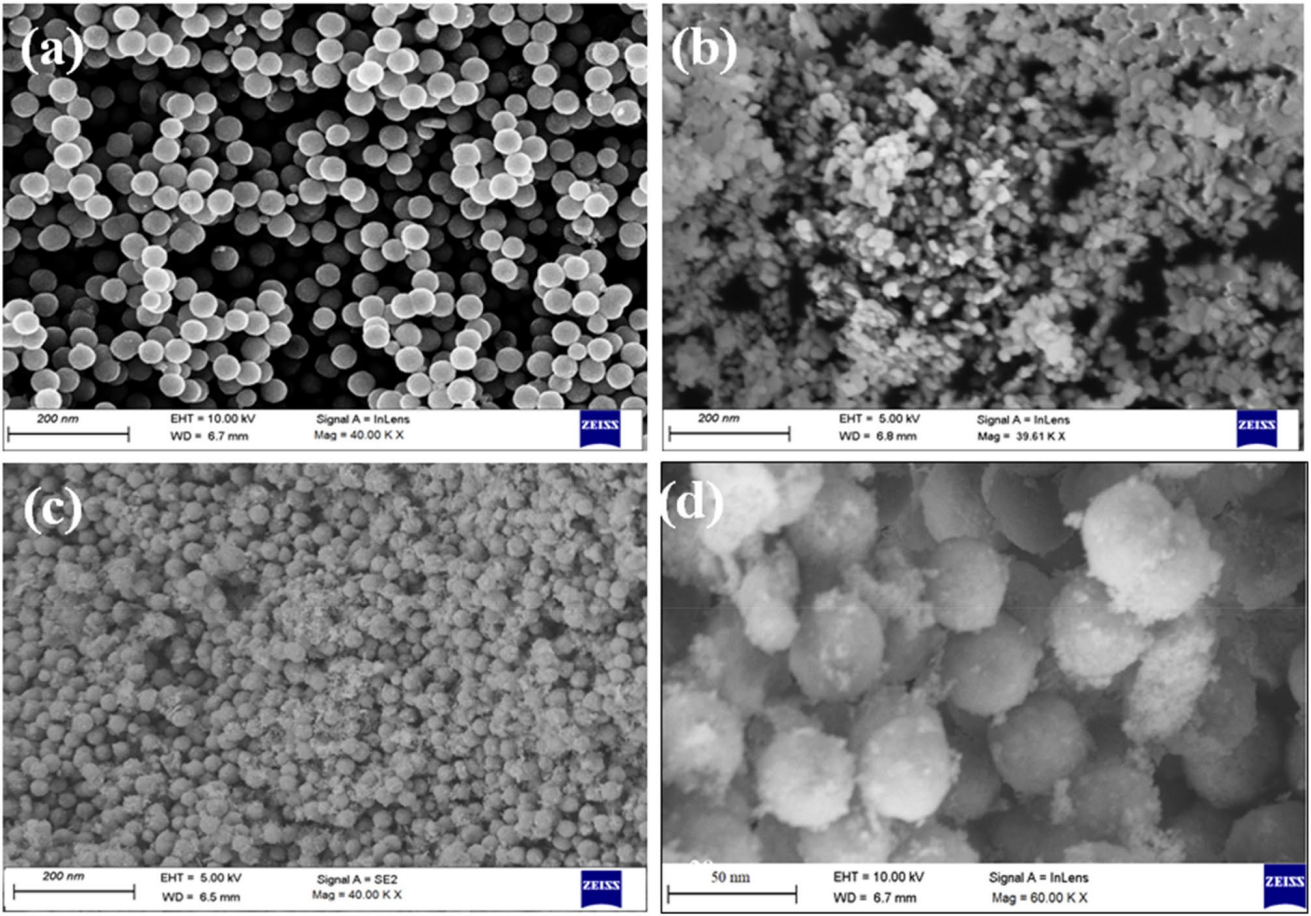
FESEM Images of (**a**) F, (**b**) N, (**c**) FN and (**d**) NF NPs.

The FESEM micrographs shown in Fig. 3 reveal the surface morphologies and the particle size of the prepared samples (a) F, (b) N, (c) FN, and (d) NF. The morphology of sample F, FN, and NF were observed as spherical, while for sample N was rod-shaped. The average particle size of the spherical sample F was observed as 40.76 ± 3 nm similar to the reported in literature^46^. The average dimensions of the rod-shaped sample N were observed as length of 34.13 ± 3 nm and width of 10.97 ± 2 nm. The rod-shaped morphology of NiO NPs has been reported by researchers and the present sample N possess similar morphology to previously reported literature^47^. For the CS samples, the observed average particle sizes were 31.75 ± 3 nm for sample FN and 36.44 ± 2 nm for sample NF. The reduction in size of CS nanostructures can be explained by diffused layers of constituents during formation of CS NPs. The sample FN and NF also showed some smaller particles residing on the surfaces, which might be attributed to the residual N NPs. All the samples showed aggregated clusters owing to the inter-particle interaction between the NPs due to the rapid increase of surface energy at the nanoscale regime as the NPs coalesce to form a stable system^48,49^.

### HRTEM

**Figure 4:**
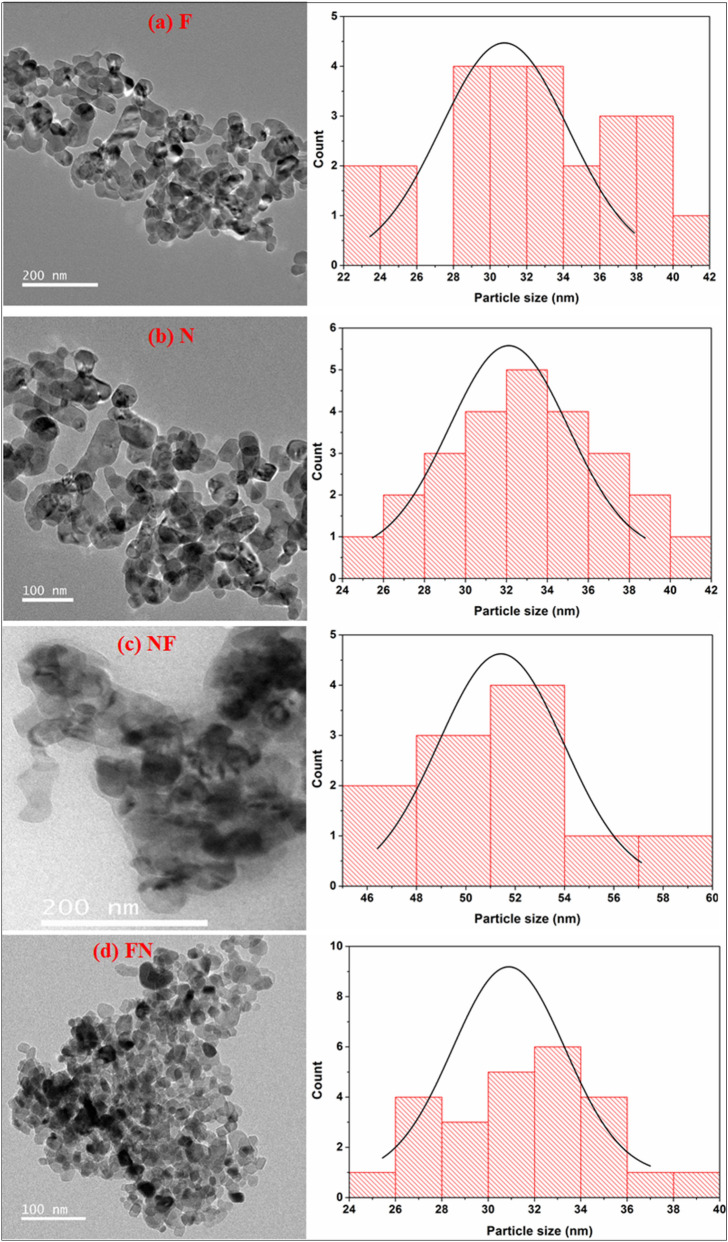
HRTEM image and particle size distribution of (**a**) N, (**b**) F, (**c**) NF and (d) FN NPs.

**Figure 5:**
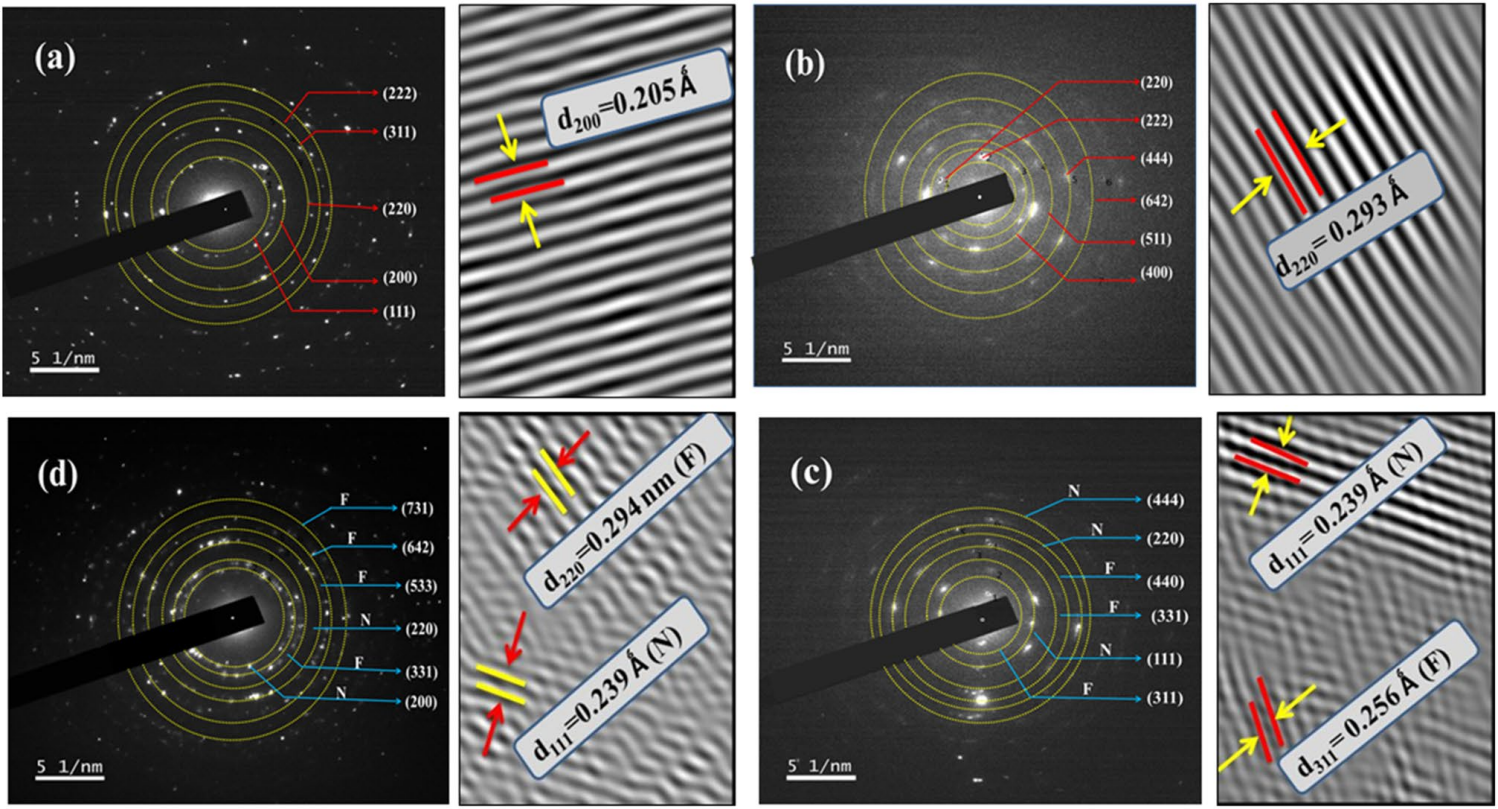
SAED pattern and lattice fringes of (**a**) N, (**b**) F, (**c**) NF and (**d**) FN NPs.

**Figure 6:**
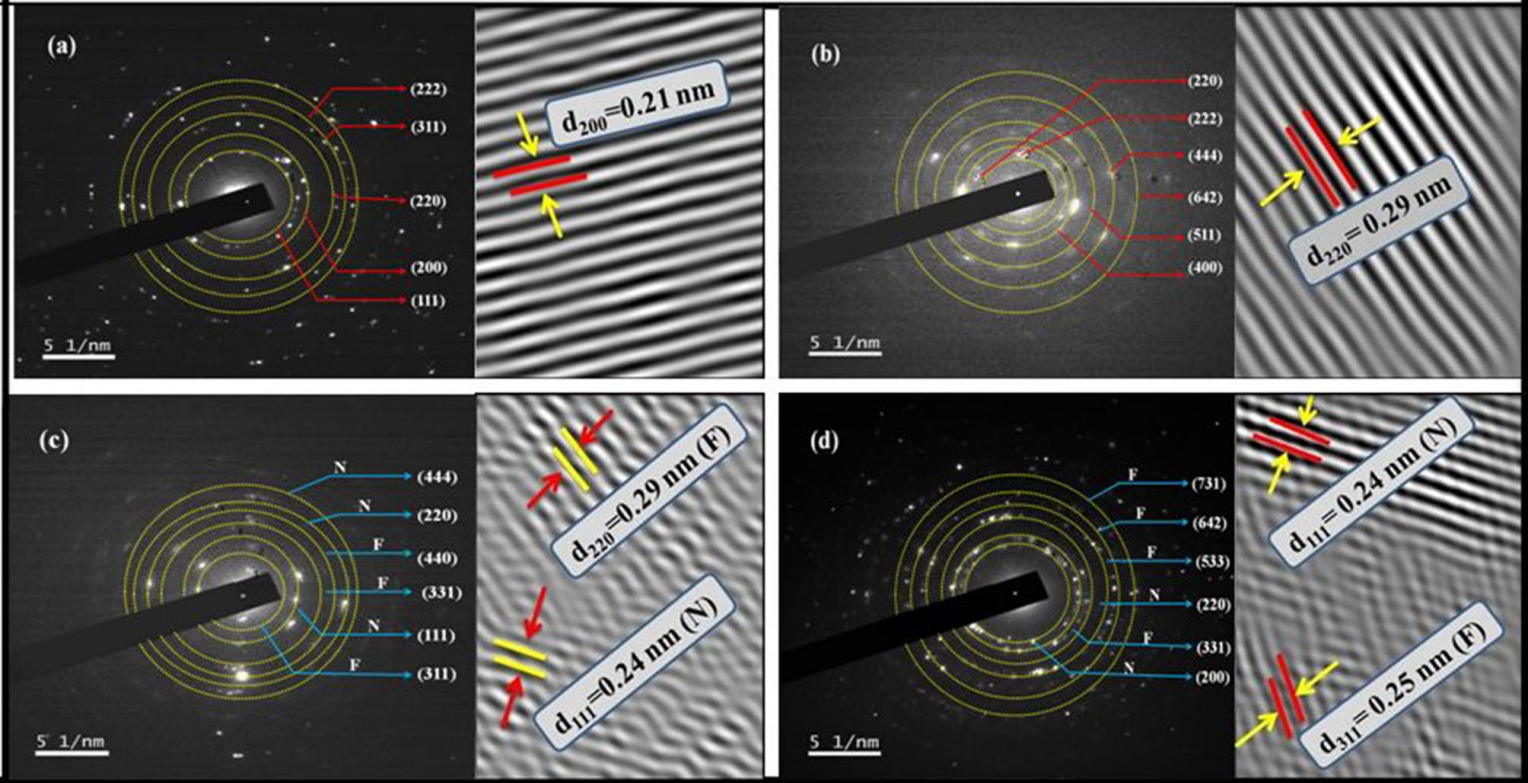
(**a**,**b**) Elemental analysis of CS nanostructures (**a**) FN and (**b**) NF.

In order to further investigate the structure, size and morphology resulted from XRD and FESEM, HRTEM analysis is performed as manifested in Fig. 4(a) N, (b) F, (c) NF and (d) FN. The TEM images clearly displayed spherical NPs. The particle sizes of both the CS systems and respective NPs (F and N) was calculated using a log normal distribution and the obtained mean particle sizes are 32.17 ± 5 nm, 32.99 ± 4 nm, 52.02 ± 4 nm and 31.55 ± 3 nm for samples N, F, NF and FN respectively. The particle sizes of the prepared NPs are found to be larger than the estimated crystal sizes (Table 1) of respective core materials. The average calculated shell thickness for NF is 2.9 ± 0.11 nm and that of FN is 3.68 ± 0.08 nm. The Inverse Fast Fourier Transform (IIFT) of the HRTEM images shown in Fig. 5a,b reveals the lattice orientation of the most intense peak (200) for N phase (d_200_ = 0.21 nm) and (220) plane for that of F sample (d_220_ = 0.29 nm). A lattice plane intersection of both N and F samples is clearly observed in Fig. 5c,d in the case of CS FN and inverted CS NF NPs. In the case of inverted CS NF, the lattice orientation of (220) planes with inter planer d-spacing of 0.29 nm reveals that the shell material is composed of F material and the lattice plane (111) with inter planer d-spacing of 0.24 nm disclosed that the core material belongs to N sample respectively. Also, for FN system, the lattice plane (311) divulge that F sample (d_220_ = 0.29 nm) is present in the core material while (111) plane belonging to N sample (d_111_ = 0.24 nm) is evident in the shell material. The concentric diffraction rings with intermittent spots captured by typical SAED pattern for samples N, F, NF and FN with polycrystalline nature is depicted in Fig. 5a–d, respectively. The electron diffraction rings reflected from sample N and F planes shown in Fig. 5a–b are consistent with the XRD results. The co-existence of both N and F planes for NF and FN systems is clearly seen in the SAED pattern (Fig. 5c–d). The diffraction rings observed due to (311), (331) and (440) reflected planes correspond to F sample and (111), (220) and (444) planes is resulted from N sample respectively for NF system. Nevertheless, for FN system, the concentric rings due to N planes are (200) and (220), while (311), (533), (642) and (444) diffracted planes are due to F sample. Figure 6a,b shows the elemental analysis of both the CS FN and NF respectively. The displayed result clearly reveals that FN and NF CS are composed of N and F materials in the core and shell. Thus, the HRTEM image displayed in Fig. 4, the SAED pattern illustrated in Fig. 5 and the elemental analysis shown in Fig. 6 distinctly affirm the emergence of CS structure for both the system and all the results are in agreement with the XRD and FESEM results.

### Magnetic measurements

**Figure 7:**
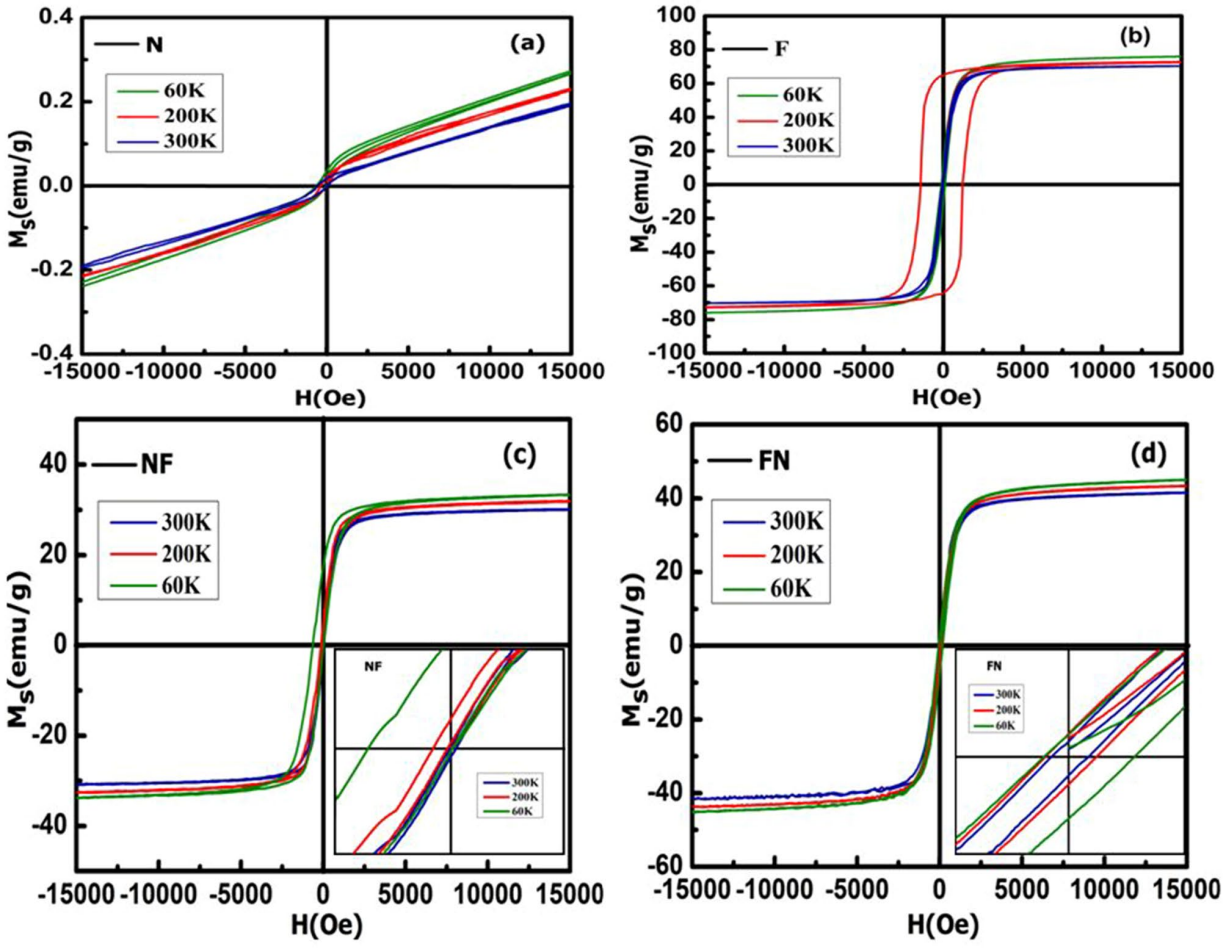
M-H loop for (**a**) N, (**b**) F, (**c**) NF and (**d**) FN NPs.

Magnetic measurements (M-H loop) are performed under the field-cooled condition at three different temperatures of 60, 200 and 300 K for N, F, NF and FN NPs as projected in Fig. 7a–d. Magnetic properties such as coercive field (H_c_), retentivity (M_r_), saturation magnetization (M_s_) and squareness ratio (M_r_/M_s_) at room temperature (300 K) are depicted in Table 2.

**Table 2:** Magnetic parameters of N, F, NF and FN NPs at room temperature (300 K).

Sample	H_c_ (Oe)	M_r_ (emu/g)	M_s_ (emu/g)	(M_r_/M_s_)
N	87.02	2.65	0.180	–
F	22.41	1.65	70.264	0.024
NF	37.09	1.35	31.33	0.043
FN	100.29	2.16	40.03	0.054

**Figure 8:**
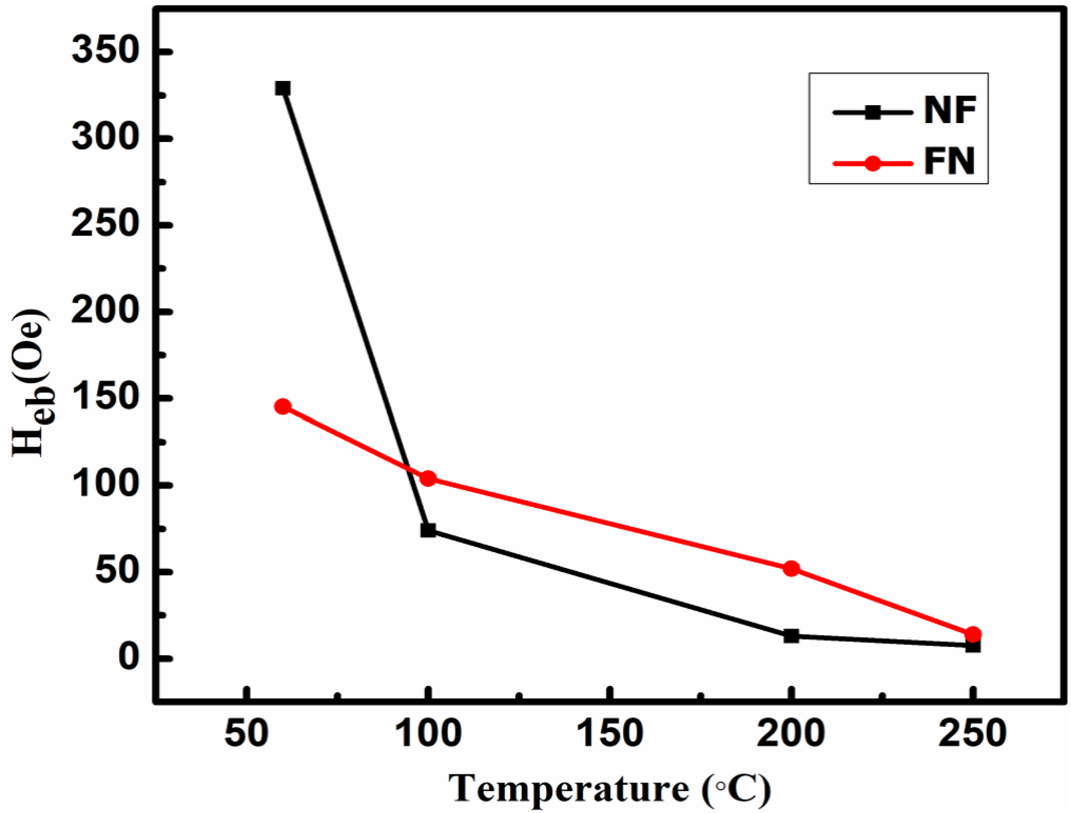
Variation of H_eb_ for CS FN and NF nanostructure with temperature.

The saturation magnetization for FN (40.02 emu/g) and NF (31.33 emu/g) are found to be lower than the bare F (70.264 emu/g) sample, indicating magnetic contributions by both the magnetic phases of N and F. The similar trend is observed for the coercivity values of CS NPs. This indicated that FN and NF are magnetically exchange coupled with each other at the interface of the two materials^13,50,51^. The saturation magnetization, as well as coercivity for inverted CS NF, is found to be lower as compared to the normal CS FN because, the maximum magnetic contribution is provided by Fe_3_O_4_ and for NF system, Fe_3_O_4_ is used as a shell with a very small thickness as compared to NiO core. Also, as indicated in Fig. 6, the saturation magnetization becomes greater with a decrease in the temperature attributing the dominance of magnetic field over temperature. Moments tend to be more aligned in the magnetic field direction and vice-versa for higher temperature^52,53^.The magnetization versus applied field recorded at 60 K for both NF and FN as shown in Fig. 7c,d perfectly exhibits an EB phenomenon with an expansion in the coercive field (H_c_) and shifting of the hysteresis loop with a field magnitude referred to as the EB field (H_eb_)^19,54^. Interestingly, the shifting of M-H loop is observed along the negative field^20,54,55^ for inverted NF with an EB field H_eb_ = − 329.43 Oe, while the M–H loop is shifted along the positive field^15,56,57^ with H_eb_ =  + 126 Oe for FN system. The variation of H_eb_ with temperature for both the CS nanostructure is shown in Fig. 8. H_eb_ at 250 K is observed to be 23 Oe for CS FN and 12 Oe for CS NF respectively. It is noticed that H_eb_ reduces with increasing temperature and becomes negligible at room temperature. It is speculated that the process of field cooling induces a unidirectional FM anisotropy at the interface which increases EB effect in both the sample^58^.The shift of hysteresis loop or EB phenomenon may be positive or negative depending upon the number of spin up (↑) or spin down (↓) uncompensated AFM pinned spins (pinning density) at the interface. Sahoo et al. theoretically showed that surface spin pinning density significantly influence the EB effect. In the ferromagnetic surface, if more number of up spin (↑) is pinned with respect to down spin (↓), then it will create the effective positive intrinsic field. To neutralize this effect, an additional negative external magnetic fields are generated resulting to a negative EB. Similarly, positive EB occurred with more pinning of down spins (↓) at the CS interface. Also, in the case of FN when shell is antiferromagnetically ordered, the spin pinning is less effective and the intrinsic field produced by the system is negligible^59^. It is well known that the amount of AFM uncompensated spins at the interface totally influence the phenomenon of the EB effect^19,60^. The rate of uncompensated spins given out over the entire nanoparticles ‘n’ and the total number of ions containing in the MNPs ‘N’ is related as $n=\frac{1}{{N}^\frac{1}{2}}$. Thus, scaling the total number of magnetic ions in NPs ‘N’ to ‘D^3^’^21^ and substituting in the above relation, the total number of uncompensated spins at the surface interface is calculated and recorded as n = 1.2 × 10^−2^ for CS FN and n = 1.5 × 10^–2^ for inverted CS NF (higher H_eb_ value) respectively.

**Figure 9:**
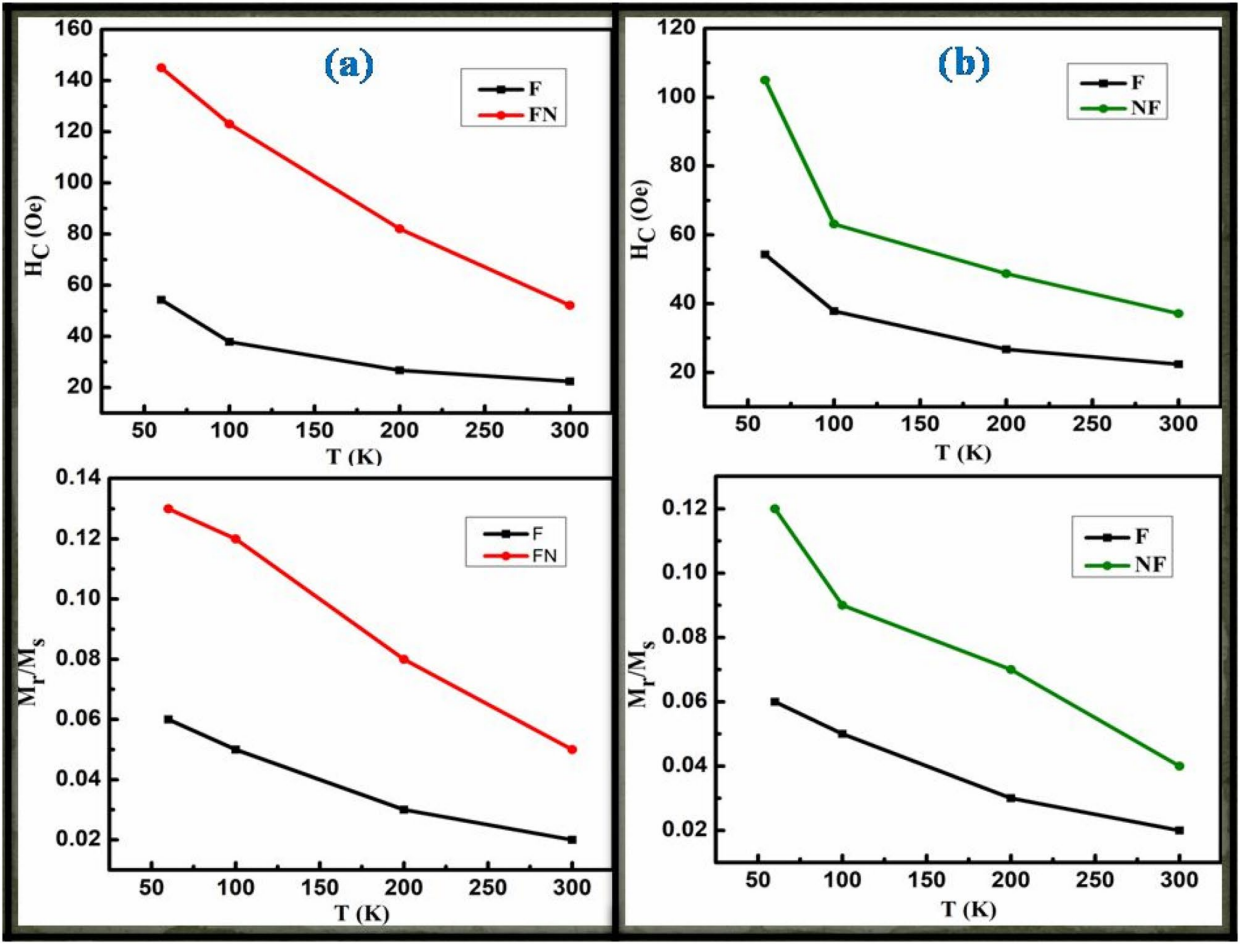
Temperature dependence of M_r_/M_s_ and H_c_ for CS (**a**) FN and (**b**) NF compared with single F phase NPs.

**Figure 10:**
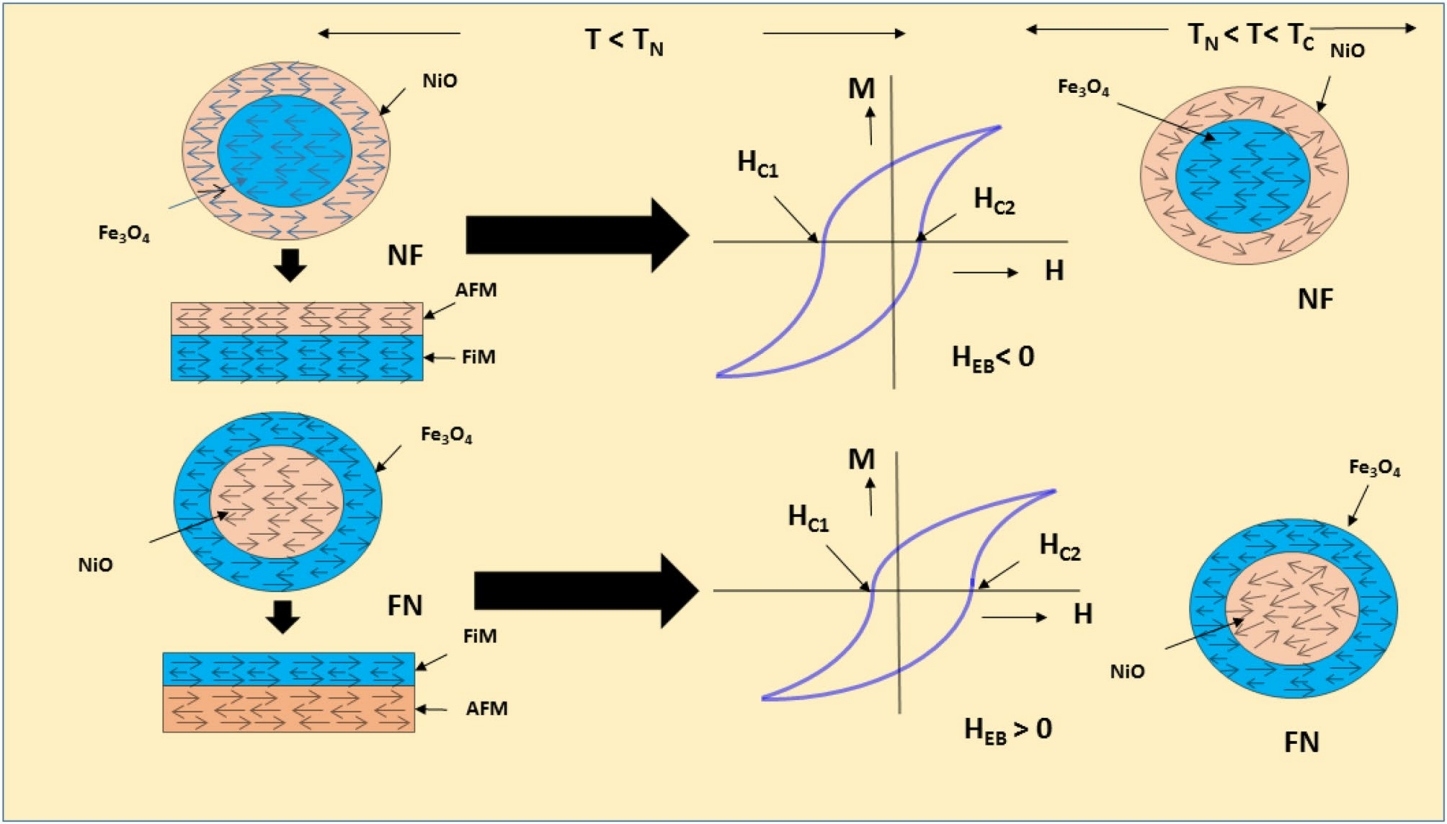
A schematic representation of negative and positive exchange bias of CS FiM-AFM and AFM-FiM NPs with spin configuration at two different temperatures of T_N_ ˂ T ˂ T_C_ and T ˂ T_N_.

Figure 9 displayed the temperature dependence of M_r_/M_s_ and H_c_ for CS (a) FN and (b) NF compared with single F phase NPs and it showed a larger harness for both the CS systems which is attributing to a strong interface coupling between the AFM and FiM phases^61^.The enhancement of H_c_ recorded at 60 K is 95.69 Oe for NF and 145.18Oe for FN, respectively. The EB phenomenon of CS FiM-AFM NPs for different stages of hysteresis loop representing the spin configuration and rotation of FiM-AFM magnetization at two different temperatures of T_N_ ˂ T ˂ T_C_ and T ˂ T_N_ under field cooling can be better understood through a qualitative schematic illustration as shown in Fig. 10. The EB field (H_eb_), H_c_ and effective anisotropy (K_eff_) for samples F, NF and FN at 60 K are tabulated in Table 3. It is well documented that anisotropy plays an important role in tuning exchange bias. The effective anisotropy of both the CS systems was calculated using the following equation ${T}_{B}=\frac{{K}_{eff}V}{25{k}_{B}}$, where k_B_ is Boltzman constant and V is the particle volume. The blocking temperature (T_B_) can be accurately measured by the temperature dependence of coercivity equation^62^ $H_{c} (T) = H_{c} (0)\left[ {1 - \left( {\frac{T}{{T_{B} }}} \right)^{2} } \right]$. It is seen that the FN system shows larger anisotropy than NF system which may be due to the increase of interface exchange coupling. The existence of EB as well as coercive field enhancement is related to the uncompensated pinned and unpinned magnetic spins of FiM-AFM and AFM-FiM at the surface interface. The amount of uncompensated AFM spins pinned to the FiM spins generates the shift in the H_eb_, while the uncompensated unpinned spins experienced an addition dragging torque generated by the FiM spins and rotates along its direction, which leads to the enhancement of coercive field^19,45,63^.

**Table 3:** K, H_eb_ and H_c_ values of F, NF and FN NPs at 60 K.

Sample	K_eff_ (× 10^4^ J m^−3^)	H_eb_ (Oe)	H_c_ (Oe)
F	5.15	–	51.8
NF	1.85	− 329.43	95.69
FN	2.33	+ 145.18	145.18

### Cytotoxicity study

**Figure 11:**
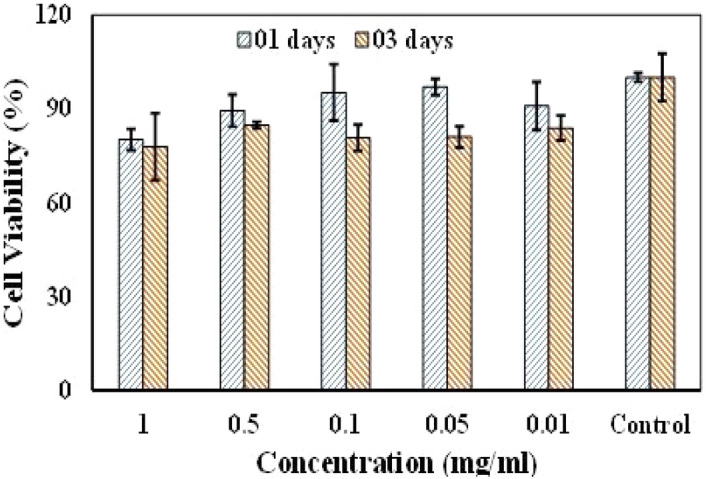
In-vitro cytotoxicity assay of FN sample incubated for 1 and 3 days using MG-63 cells.

Cytotoxicity of MNPs is one of the most important keys to undergo magnetic fluid hyperthermia (MFH) and also for other biomedical oriented treatment. Figure 11 shows the cytotoxicity assay for one of the synthesized samples, FN MNPs using MG-63 cells incubated for 24 and 72 h at five different sample concentrations (0.01, 0.05, 0.1, 0.5 and 1 mg/ml) at 37 °C in 96 well tissue plates. As depicted in Fig. 11, the FNMNPs are proven to be nontoxic as guided by the biological evaluation for in vitro cytotoxicity test of part 5^64^, as the cell viabilities are above 70% at all different concentrations for upto 72 h respectively. However, as observed usually in biocompatibility test, reduction in the cell viability percentage is manifested with increasing the sample concentration and incubation time^37,39,65^. Therefore, based on the cytotoxicity response, FN sample is evidently nontoxic even at 1 mg/ml concentration incubated for 72 h, which marks the samples as biocompatible and thus nanocomposites of NiO and Fe_3_O_4_ are suitable for magnetic fluid hyperthermia (MFH) treatment.

### Induction heating study

**Figure 12:**
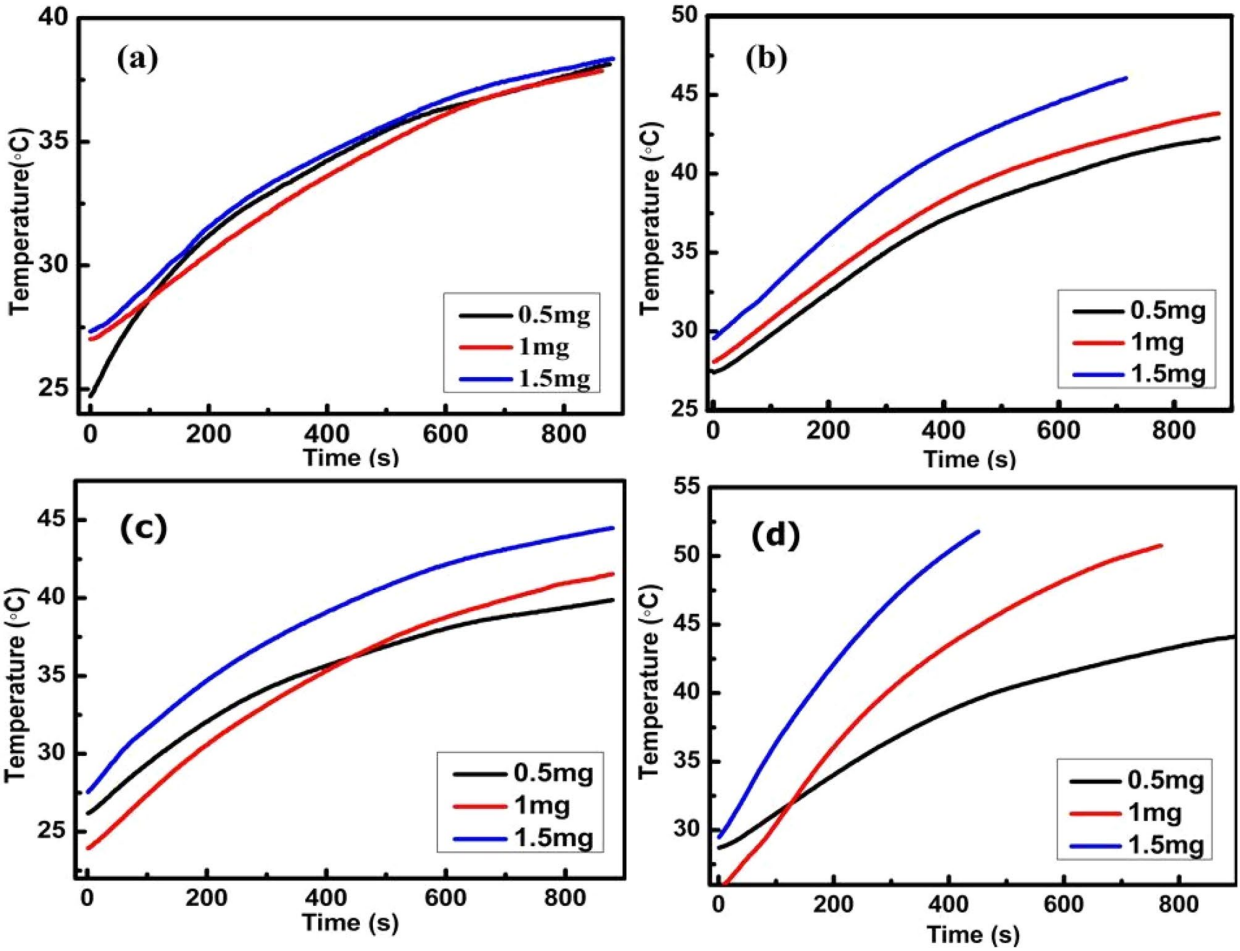
Temperature versus time curves for (**a**) N, (**b**) F, (**c**) NF and (**d**) FN NPs at field amplitude *H* = 9.24 kAm^−1^ and frequency *f* = 337 kHz.

Figure 12 displays the self heating characteristics of N, F, NF and FN NPs at three different concentrations of 0.5, 1 and 1.5 mg at a frequency *f* of 337 kHz and field amplitude *H* of 9.24 kAm^−1^, which are within the clinical limits^6^. F, NF and FN MNPs reached to the hyperthermia temperature of 44 °C within 10 min. Synergistic effects are observed in both NF and FN system. FN samples achieved hyperthermia temperature at a concentration of 1 mg, while the same is attained at 1.5 mg in case of NF samples because of lesser content of Fe_3_O_4_ in the shell. The SAR value was extracted from the initial slope of temperature versus time graph up to 900 s and it has shown dependency on the concentration. The SAR values are obtained using the formula given by $SAR=C\times \frac{dT}{dt}\times \frac{{m}_{a}}{{m}_{s}}$, where C is the specific heat capacity of the solvent, $\frac{dT}{dt}$ is the initial slope, ${m}_{a}$ is the mass of the solute plus mass of solvent and ${m}_{s}$ is the mass of solute^66^. Since the power dissipated by any given MNPs scales quadratically with the applied magnetic field strength and linearly with the frequency, one must normalise the respective SAR values by removing these extrinsic properties which are determined by the intrinsic loss parameter (ILP) given by the expression $ILP=\frac{SAR}{{fH}^{2}}$^67^.The respective SAR and ILP values for all the samples at three different concentrations are listed in Table 4. The maximum calculated SAR values obtained are 167, 216, 232 and 278 W/g for N, F, FN and NF, respectively at 0.5 mg concentration and it reduces as we further increase the sample concentration. The probable reason for the reduction of SAR value with increase in concentration is due to the dipolar interaction during the spin relaxation process. Generally, dipolar interaction energy imposes a disordering torque which interrupts the Néel relaxation of the magnetic moment through the energy barrier^68^. Interestingly, despite the higher values of saturation magnetization and anisotropy for F, the NPs heating response is more positive or higher SAR is obtained for CS nanocomposites FN and NF, which is due to the magnetic EB or exchange coupling at the interface of the two materials^12,26,29^. It indicated that the interface exchange anisotropy of the CS arrangements tuned the effective anisotropy, which in turn enhanced the heating efficiency of the MNPs^6^. Khurshid et al. concluded that cubical particles showed more heating efficiency than spherical particles of similar nature, which attribute that the surface anisotropy plays an important role in improving heating efficiency^69^.Therefore, the higher surface anisotropy of Fe_3_O_4_ shell with higher EB field for NF system plays the key role in enhancing the SAR value as compared to FN system^6^.

**Table 4:** SAR and ILP values at three different concentrations for N, F, NF and FN samples.

Samples	SAR (W/g)			ILP × 10^–3^ (nH m^2^ g^−1^)		
	0.5 mg	1 mg	1.5 mg	0.5 mg	1 mg	1.5 mg
N	167	76	63	5.8	2.6	2.1
F	216	117	87	7.5	4.1	3.2
NF	278	150	119	9.7	5.2	4.2
FN	239	202	186	8.3	7.1	6.5

In addition, it is also seen that the SAR value at higher concentration shows different trend, i.e. the FN system has higher SAR value over the NF system. Silva et al*.* have experimentally demonstrated that the EB effect is hindered or lowered at higher concentration due to more dipolar interactions, which plays as a demagnetizing role originating from the anisotropic nature of the magnetic dipole–dipole fields^70^. Since F is used as the shell material for inverted CS NF, the MNPs are magnetically more attracted leading to more agglomeration of particles because of more dipolar interactions as compared to the usual FN system where N is used as the shell material. Now, since NF particles are magnetically more attracted, increasing in the sample concentration enhances dipolar interactions which hinder or decrease the EB coupling and therefore, reduction of SAR values are observed as compared to FN samples. Thus, based on the heating response, it is evident that EB effect produces due to exchange coupling of two different materials at their interfaces leads to enhancements of the heating capability of MNPs. The greater EB effect results in the better heating capacity of the MNPs. Therefore, nanocomposite of normal CS FN and inverted CS NF are more efficient than pristine NPs and are suitable for magnetic fluid hyperthermia (MFH) applications.

## Conclusions

A two-step synthesis process involving the co-precipitation method and solvothermal process has been used to fabricate an inverted CS (NiO@Fe_3_O_4_) and usual CS (Fe_3_O_4_@NiO) nanostructures. A systematic investigation on their magnetic properties and microstructure were examined through VSM, XRD, FESEM and HRTEM. XRD and HRTEM analyses confirmed the phase purity and core–shell formation of N and F materials. Magnetic measurements at 60 K have displayed a perfect negative EB effect (H_eb_ = − 329.43 Oe) for inverted CS system, while a positive EB effect (H_eb_ =  + 145.18 Oe) for the usual CS counterparts. The cytotoxicity profile of Fe_3_O_4_@NiO NPs clearly indicated is the non toxic characteristics for 72 h of incubation even at higher sample concentrations up to 1 mg/ml. The self heating responses for CS nanocomposites FN and NF have recorded higher SAR values over the single-phased F, despite having lower saturation magnetization and magnetic anisotropy. The interface exchange anisotropy and surface anisotropy of CS nanostructures enhanced the SAR value. Also, inverted CS NF with greater EB coupling and more surface anisotropy contribution from F shell have shown higher SAR value in comparison to normal CS FN. Thus, EB effect or interface exchange anisotropy and surface anisotropy positively influence the heating ability of the MNPs. Therefore, normal CS FN and inverted CS NF can be applied for magnetic fluid hyperthermia applications.
