# Publisher Correction: A comparative investigation of normal and inverted exchange bias effect for magnetic fluid hyperthermia applications

**DOI:** 10.1038/s41598-021-83029-y

**Published:** 2021-02-03

**Authors:** S. P. Tsopoe, C. Borgohain, Rushikesh Fopase, Lalit M. Pandey, J. P. Borah

**Affiliations:** 1 Department of Physics, National Institute of Technology Nagaland, Dimapur, Nagaland 797103 India; 2 Central Instrumentation Facility (CIF), Indian Institute of Technology Guwahati, Guwahati, 781039 India; 3 Bio-Interface & Environmental Engineering Lab, Department of Biosciences and Bioengineering, Indian Institute of Technology Guwahati, Guwahati, Assam 781039 India

Correction to: *Scientific Reports* 10.1038/s41598-020-75669-3, published online 29 October 2020

This Article contains an error in Figure 5 where the values for the d-spacing are incorrect. The correct Figure 5 appears below as Figure 1.

**Figure 1:**
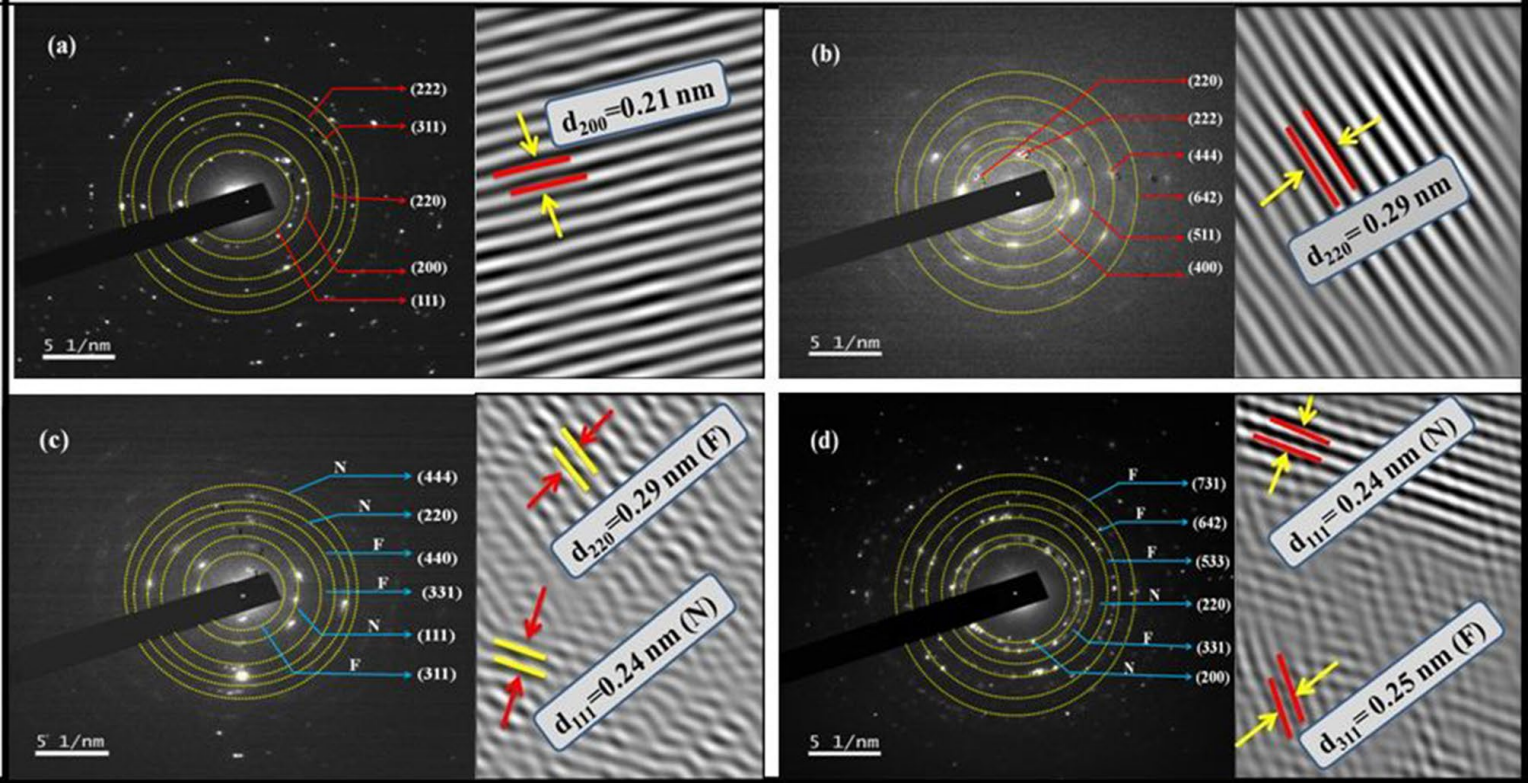
SAED pattern and lattice fringes of (**a**) N, (**b**) F, (**c**) NF and (**d**) FN NPs.

Additionally, Figure 6 is a duplication of Figure 5. The correct Figure 6 appears below as Figure 2.

**Figure 2:**
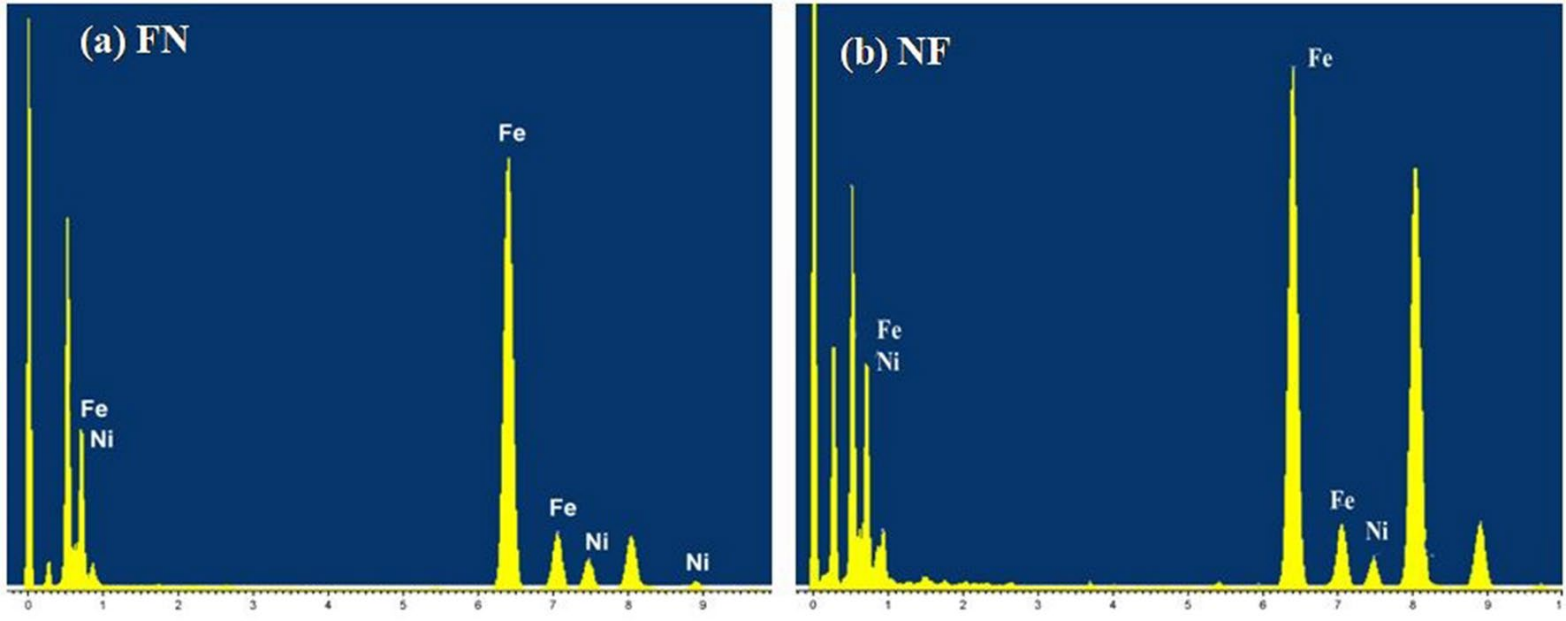
(**a,b**) Elemental analysis of CS nanostructures (**a**) FN and (**b**) NF.

